# Double-Edged Sword Effect of High-Performance Work System on Employee Well-Being—Moderating Effect of Union Practice

**DOI:** 10.3389/fpsyg.2021.619345

**Published:** 2021-08-05

**Authors:** Wei Qi, Hu Enhua, Sun Jiandong, Shan Hongmei

**Affiliations:** ^1^School of Management, Jinan University, Guangzhou, China; ^2^School of Economics and Management, Nanjing University of Aeronautics and Astronautics, Nanjing, China; ^3^School of Management, Guilin University of Aerospace Technology, Guilin, China; ^4^School of Management of Nanjing University of Posts and Telecommunications, Nanjing, China

**Keywords:** employee well-being, high-performance work system, union Practice, perceived organizational support, work stress

## Abstract

Improving the well-being of the employees is the inevitable choice to improve corporate performance and competitive advantage and the social responsibility that enterprises must undertake. Based on the job demands-resources model, this study introduces perceived organizational support and work stress as the mediator and trade union practice as the moderator to explore the double-edged sword effect of a high-performance work system (HPWS) on the well-being of the employee. Taking 243 employees from Jiangsu, Zhejiang, and Anhui as samples, we found that HPWS positively affects the well-being of the employee through perceived organizational support and negatively affects the well-being of the employee through work stress. Union practices can significantly reduce the positive effect of HPWS on work stress and further weaken the negative effect of HPWS on the well-being of the employee through work stress. The results of this study provide a new way to explain the impact of the HPWS on the well-being of the employees and find that union practice can weaken the negative effects of HPWS. This study provides a new thinking direction for improving the well-being of employees in enterprises.

## Introduction

The well-being of the employee refers to the quality of the whole experience of employees in the workplace (Grant et al., 2007). High-level well-being of employees reduces turnover tendency (Page and Vella-Brodrick, 2009), increases work performance (Robertson et al., 2012), and creativity of the employees (Miao and Cao, 2019). Therefore, improving the well-being of the employee remains an essential topic for enterprises to gain a competitive advantage and to sustain their development.

There is a close relationship between human resource management (HRM) practices and the well-being of the employee in organizations, especially the high-performance work system (HPWS), which is regarded as “the best HRM practice” (Ho, 2018). Initial research believed that HPWS provides employees with the necessary resources to help them complete the work and accomplish career development, thus increasing the well-being of the employees (Zhang et al., 2013); however, the frequent emergence of management accidents like “the sudden death of programmer in China” provoke a rethinking of people of the current management systems in enterprises (Jiandong et al., 2020). Some studies also criticize the adverse side of HPWS caused by the high job requirement embedded in it (Van De Voorde and Beijer, 2015; Liu et al., 2020).

To find ways to reconcile the dark side of HPWS, it is essential to understand the internal mechanism of how HPWS influences the well-being of the employee first. Although several studies proposed that there may be a double-edged sword effect of HPWS on the well-being of the employee (Han et al., 2020), there is no comprehensive framework to explain the complex relationship between HPWS and the well-being of the employee. Fortunately, the job demands-resources model (JD-R) provides a framework to explain the double-edged sword effect of HPWS on the well-being of the employee. According to the JD-R model, job resources nourish the well-being of the employees; job demands cause the loss of resources and threaten resources loss, which reduces the well-being of the employee (Bakker and Demerouti, 2007). Current studies show that the resources of HPWS can help employees improve their skills and accumulate capital for their career development, thus making them perceive more organizational support (Zhong et al., 2016). Therefore, perceived organizational support may be a “resource path” of HPWS to affect the well-being of the employee positively. Demerouti et al. (2001) argued that job demands accelerate resource consumption or make employees perceive the threat of resource loss, immediately leading to increased work stress. Therefore, this study selects work stress as the “demand path” to understand how HPWS affects the well-being of the employee negatively.

High job demand inevitably coexists with job resources in the HPWS because of the profit orientation of enterprises. Therefore, utilizing the power of other organizations may be an effective way to reconcile the dark side of HPWS and builds a fair and effective management system in the workplace (Guest, 2017). Trade unions are always regarded as facilitators to HRM through assistance and supervision in enterprises (Cook et al., 2020). For example, Chen et al. (2018) found that union practice can create a harmonious labor-management atmosphere in the workplace, which improves the impression of the employees on the enterprise, thus strengthening the positive relationship between HPWS and the positive experience of employees. Bryson et al. (2013) found that social support of trade unions weaken the relationship between transformative HRM practices and anxiety of employee by providing essential support.

Based on existing research and combined with the JD-R model, this study employs perceived organizational support and work stress as mediating variables to understand the effect of HPWS on the well-being of the employee. At the same time, it introduces union practice as a moderating variable to explore the influence of union practice on the relationship between HPWS and the well-being of the employees.

## Theory and Hypotheses

### HRM and Union in China

Confronted with great changes in social, cultural, legal, and economics from the “reform and opening” in 1979, many Chinese enterprises have come across difficulties in survival and development (Min et al., 2018). Therefore, Chinese enterprises began to implement the HPWS which is aligned with the recommendation of western scholars, putting high strategic importance on selection, training, performance appraisal, participation, and information sharing (Chen et al., 2018). It has been reported that the majority of foreign multinational corporations, state-owned enterprises, and big private enterprises have adopted HPWS (Cooke, 2004).

From 2010, with the promulgation of the three labor laws (Labor contract Law, Employment Promotion Law, and Labor dispute Mediation and Arbitration Law) in China, the Chinese government accelerated the pace of establishing unions in enterprises, the density of unions, and the number of union members has reached an unprecedented zenith (Cooke, 2014). Chinese unions are always embedded in the enterprises and existing as a department in the enterprise (Liu and Li, 2014). Therefore, trade unions are the organizations that directly face employees in the enterprise, and there is a non-negligible influence of union on the HRM (Snape and Chan, 2018) and given that the Chinese union undertakes two main responsibilities, protecting the right and interests of employees and promoting the work efficiency of enterprises, it always acts as supervisor and assistant of HPWS simultaneously (Budd et al., 2014). For example, Wang and Lien (2018) found that the practice of trade unions can effectively increase the overall wage level of employees. Lee et al. (2016) found that trade unions can enhance the effectiveness of HRM by improving the atmosphere in the workplace or by transmitting information about employees.

### Application of JD-R Model

Demerouti et al. (2001) first proposed the JD-R model. After nearly 20 years of testing, it has become one of the classic organizational behavior theories in explaining employee attitude and behavior. In this study, the JD-R model provides a framework to understand the relationship between HPWS and the well-being of the employee. The JD-R model hypothesizes that all work characteristics can be divided into job resources and job demands (Demerouti et al., 2001). Job resources have the nature of motivation, which leads to positive organizational results. In contrast, job demands increase the resource consumption of employees and trigger negative consequences, such as burnout and emotional exhaustion (Bakker and Demerouti, 2007). In the JD-R model, job resources refer to everything that helps employees achieve their goals, including work autonomy, performance feedback, social support, and superior guidance (Bakker and Leiter, 2010). For employees, the practices in HPWS, such as training, job autonomy, salary fairness, promotion channels, support them to acquire promotion, increase their salary, and realize their value. Employees will regard these practices as the symbols, which represent the concern and support from organization (Zhong et al., 2016). Therefore, in this research, we select perceived organizational support as the “resource path” through which HPWS affects the well-being of the employee positively. Job demands refer to all objects that consume resources or may cause resource losses in the JD-R model (Bakker and Leiter, 2010). The ultimate goal of HPWS is to help enterprises gain more profit, so the premise of enjoying the resources provided by HPWS is that employees can meet the job demands proposed by it. A significant reflection is that promotion and increased salary are always linked with work performance (Dai et al., 2018). To protect resources they hold in organizations, employees must invest time, energy, and other personal resources to prevent the loss of resources, such as salary and promotion opportunities. The gap between resources the employees need to invest and resources they hold will cause increased work stress immediately (Hobfoll et al., 2018). Therefore, in this study, we select work stress to understand the “demand path” through which it influences the well-being of the employee negatively.

The JD-R model also points out that the perception of employees on job demands and job resources is determined by various environmental factors (Bakker and Demerouti, 2007). First of all, resources are contextual, the perception and evaluation of resources by employees are always associated with the environment (Bakker and Demerouti, 2018). In recent years, with the progress of trade union reforms, trade unions in China have begun to play an important role in enterprises (Chen et al., 2018). In the enterprise, the Chinese union needs to protect the rights and interests of employees and undertake the responsibility of improving the cultural achievement of employees and enriching their spiritual lives. Therefore, unions provide additional knowledge supplements for employees and promote work skill exchange among them through skills competitions, knowledge quizzes, and other activities (Chung, 2016). These activities enable employees to make better use of the resources provided by HPWS, which may increase perception of the value of such resources by employees. Second, the work stress of employees mainly depends on the judgment of employees on the loss of resources (Bakker and Demerouti, 2018). As a protector of rights and interests of employees, Chinese unions help employees translate their grievances and requirements to the enterprise through collective agreements, collective voices, and other practices and protect their fundamental rights and interests (Budd et al., 2014), which can reduce judgment of employees on resource loss. Therefore, we hypothesize that the practice of the union can moderate the relationship between HPWS and organizational support and between HPWS and work stress.

### Perceived Organizational Support as a Mediator

A high-performance work system refers to a series of management practices to improve the performance of the company, mainly including practices such as recruitment and selection, performance compensation, training, employee participation, and information sharing (Sun et al., 2007). Eisenberger et al. (1986) argued that proper promotion, comprehensive training, and job richness make employees perceive more organizational support. Therefore, it can be seen that HPWS can nourish perceived organizational support of employees which refers to the subjective evaluation of employees of concern of the organization about their welfare and contribution (Eisenberger et al., 1986). First, comprehensive training for employees can improve their work skills and accumulate capital for their career development (Wayne et al., 1997). Second, practices, such as employee participation, training, and internal promotion, in the HPWS can help employees identify and use resources in the workplace better to achieve their goals (Kuvaas, 2008). As a consequence, employees may consider that they have received the attention and support of the enterprise. Third, practices, such as employee participation and work autonomy, in the HPWS convey the signal to employees that enterprise regards them as partners and are willing to invest long-time in them, which makes them feel that the company values their development and contributions (Van De Voorde et al., 2012).

Perceived organizational support could meet the psychological needs of employees and improve the impression of the organization among employees. First, when employees perceive high organizational support, they will think that they have gained the attention of the organization through hard work and contributions. This relationship can satisfy their emotional needs in the enterprise (Eisenberger et al., 1986). Second, employees with high perceived organizational support hypothesize that they have enough resources to complete work or solve the difficulties. This reduces adverse experiences of employees, such as tension and anxiety (Eisenberger and Stinglhamber, 2011). Third, resources can improve the impression of the enterprise among employees. When perceived organizational support is high, employees think that the activities of the enterprise are based on consideration of their well-being and interests, rather than the interests of employers, which will make employees generate more positive feelings (Gillet et al., 2012). In summary, perceived organizational support can encourage employees to have more positive experiences and reduce negative experiences, such as anxiety and emotional exhaustion, improving the well-being of the employees. Therefore, this study proposes the hypotheses:

H1: Perceived organizational support mediates the positive relationship between HPWS and the well-being of the employee.

### Work Stress as a Mediator

Work stress refers to the subjective perception of the individual of the gap in how much resources they can get and how much they should invest to meet the demands and the possibility of negative consequences of such a gap (Selye, 1976). In enterprises, the resources provided by HPWS are often accompanied by high job demands (Jensen et al., 2013), which may lead to increased employee stress. Compared with traditional HRM practices, the HPWS emphasizes giving employees more participation and autonomy. This authorization also comes with independent decision-making responsibilities so that employees perceive higher job demands (Ramsay et al., 2000). Second, the HPWS links salary and promotion with performance. This requires employees to improve their work efficiency and put more time and energy into work to ensure their competitiveness in the enterprise, which increases the appraisal criteria perceived by employees (Han et al., 2020). Although the HPWS increases performance evaluation standards of employees, it does not emphasize quantitative work or set clear goals. Employees must invest as much energy as possible to get a better appraisal, further increasing the job demands they perceived (Chaudhuri, 2009). When employees face high job demand, they perceive that the resources invested (such as working hours and energy) are insufficient to meet high job demands. This gap makes them foresee the loss of resources, such as reduced wages and blocked promotions, resulting in higher work stress (Jamal, 1999).

When facing work stress, employees will invest more time and energy to prevent resource loss in the workplace. Their emotional, physical, and other personal resources will be consumed quickly, resulting in anxiety and fatigue, reducing their experienced quality (Holroyd and Lazarus, 1982). Besides, long-term work stress will make employees think that the enterprise does not provide them with the corresponding support to complete the work, which will lead to disappointment, frustration, and other bad experiences (Hakanen et al., 2008). Empirical research results also show that work stress will reduce the well-being of the employees. For example, Siu (2002) found that the positive emotional experience of employees will decrease when employees face high work stress. Golparvar et al. (2012) found that when employees face tremendous work stress, their psychological resources will be quickly consumed, leading to adverse experiences, such as anxiety and emotional exhaustion. Liu et al. (2020) found that work stress can significantly reduce the positive experiences of employees, such as job satisfaction in the enterprise. Therefore, this study proposes the hypotheses:

H2: Work stress mediates the relationship between HPWS and employee well-being.

### Union Practice as a Moderator

Halbesleben et al. (2014) pointed out that perception of resources by employees and resource value from an object will be affected by environmental factors. In different environments, employees have different sensitivity and value judgments to the same resources. In the workplace, union practices, which refers to practices carried out by trade unions to perform their duties, are essential components of the workplace environment (Mao-long et al., 2018). Trade unions guarantee employees to participate in enterprise affairs by convening employee representative meetings, establishing employee committees (Wang, 2016). In addition, when employees encounter unfair treatment in the workplace, trade unions also represent employees to bargain with the enterprise. These practices can create a fair atmosphere in the workplace (Cooke et al., 2016). Under a fair atmosphere, employees may perceive that they can reasonably utilize the resources in the workplace, such as promotion channels, information sharing, training, to achieve their goals. Employees are more sensitive to such resources, resulting in higher perceived organizational support (Ambrose and Schminke, 2003). Furthermore, there is a matching effect between resources. When the resources provided enable an individual to make better use of the existing resources, the value judgment of the individual and sensitivity to the existing resources will increase significantly (Hobfoll et al., 2018). An important duty of the Chinese union is to improve the employability of the employees, so it always holds experience-sharing meetings and provides a voluntary training course for employees (Mao-long et al., 2018). These practices may make employees more adapted to utilize resources provided by HPWS, such as work autonomy and work participation. It will increase the evaluation and sensitivity of employees to such resources. As a consequence, employees perceive often that the organization provides them with essential resources and concern their contribution and welfare. Therefore, the study proposes the following hypotheses:

H3: Union practice will moderate the relationship between HPWS and perceived organizational support, such that this relationship is stronger when union practice is high.

The work stress of employees mainly comes from the predicted loss of resources or the threat of resource loss caused by job demands. Therefore, their work stress can be effectively relieved when they can obtain related resources to cope with job demands or the assessment of resource loss is reduced (Hobfoll, 1989). As the protector of rights and interests of employees, it is union's duty to transmit employees' grievance to the enterprise and establish an executive committee to resolve daily conflict (Cooke et al., 2016). These practices make employees perceive that they can get support from the union when they confront the high job demand. In addition, unions sign collective labor contracts on behalf of employees with enterprise, which guarantees employment tenure of employees, working hours, and wage levels (Budd et al., 2014). These practices reduce the evaluation of resource losses by employees when they cannot fulfill high requirements. Therefore, when the union practices are high, the high job demands of HPWS will not cause high work stress due to additional ways to reduce resource loss and low resource loss evaluation. When the practice of the labor union is poor, they will perceive a greater loss of resources or threats of resource loss (such as overtime, reduced salary, and blocked promotion) caused by high job demands brought by HPWS, which cause high work stress. Therefore, the study proposes the following hypotheses:

H4: Union practice will moderate the relationship between HPWS and work stress, such that this positive relationship will be weaker when union practice is high.

According to hypothesis H1 and hypothesis H3, and the relationship revealed by hypothesis H2 and hypothesis H4, we further propose two moderated mediation hypotheses. Specifically, perceived organizational support mediates the positive effect of HPWS on the well-being of the employee, but this mediating effect will be affected by union practices. When union practice is high, the HPWS has a greater impact on perceived organizational support. Therefore, perceived organizational support transmits a more positive effect of the HPWS on the well-being of the employee. On the contrary, when the union practice is low, HPWS has less influence on perceived organizational support. At this time, the HPWS has a less positive impact on the well-being of the employee. Work stress mediates the negative impact of HPWS on the well-being of the employee, and the mediating effect is also affected by union practice. When the union practice is high, the HPWS has less impact on work stress, so work stress transmits less impact of HPWS on the well-being of the employee. On the contrary, when union practice is low, HPWS has a greater impact on work stress. At this time, work stress transmits a more negative impact of HPWS on the well-being of the employee. Therefore, this study proposes two moderated mediation hypotheses:

H5: Union practice will moderate the relationship between HPWS and the well-being of the employee through perceived organizational support such that this indirect effect will be stronger when union practice is high.
H6: Union practice will moderate the relationship between HPWS and the well-being of the employee through work stress such that this indirect effect will be weaker when union practice is high.

## Method

### Sample

We collected survey data from enterprises with the union in China through a paper-pencil survey from July 2020 to September 2020. First, we recruited voluntary HRM managers with an MBA degree from the university and chose those whose enterprise has built the union. Eventually, 16 HRM managers were willing to help us recruit participants and organized the survey. These enterprises are located in Zhejiang, Jiangsu, and Anhui province in China. Consequently, we visited these enterprises and conducted the survey. To ensure the quality of the survey, we promised that the data we collected will only be used in academic research to participants and assured them that the questionnaire is without identical information. In addition, the administrative staff was requested to leave the room when the participants were filling their questionnaires.

According to Bartlett et al. (2001), when we decided that the reliability of the sample is 95%, the error is 3%, and the range of variance is 3 times the SD around mean, the sample should be around 200. We also decided to collect 50% more samples to avoid a shortage of samples caused by deleting invalid cases. Therefore, we distributed 300 questionnaires, and 282 questionnaires were returned from participants. Then we deleted the inalid questionaires through two ways: one is to delete the invalid questionaires by the answer of detect items, and another is to delete questionaires that contain six or more consecutive identical items. Finally, 39 questionnaires were deleted, and 243 questionnaires were retained. The basic information of the valid sample was as follows: men accounted for 47.3%, and women accounted for 52.7%. In terms of age, 14.4% belonged to 20–25 years old, 39.1% were 25–35 years old, 22.2% were 35–45 years old, and 24.3% were over 45 years old; from the perspective of political status, party members accounted for 22.2%, non-party members accounted for 77.8%; from the perspective of the nature of the company, private enterprises accounted for 45.3%, central enterprises or state-owned enterprises accounted for 17.3%, foreign-funded enterprises accounted for 13.6%, and other enterprises accounted for 23.9%; from the perspective of company size, <100 employees accounted for 43.2%, 100–300 employees accounted for 18.5%, 100–300 employees accounted for 11.5%, 1,000 employees or more accounted for 26.7%; from the perspective of position, senior management of the company accounted for 6.6%, the middle-level leaders accounted for 16.5%, grassroots leaders of the company accounted for 17.7%, and the grassroots employees accounted for 59.3%.

### Measures

In this study, all variables are measured using popular scales which are frequently used worldwide. Among them, the English scale has been revised to Chinese using translation and back-translation procedures. All scales were measured using a 5-point Likert scale, with 1 point representing strong disagreement and 5 points representing strong agreement with the description of the question.

#### High-Performance Work System

The HPWS scale adopted the scale developed by Junwei et al. (2017), which contains all the core practices of HPWS, including 7 dimensions: comprehensive recruitment, strict selection, extensive training, development-oriented performance management, performance compensation, flexible work system, and decision-making and information sharing. The sample items include “The company will continue to provide me with training,” “My salary and bonus are determined by my performance.” The scale includes 18 items, and the Cronbach's α coefficient of the scale is 0.93.

#### Perceived Organizational Support

The scale of perceived organizational support was adapted from Eisenberger et al. (1986), including 6 items. The sample items include “The company cares very much about me” and “The company is happy to help me.” In this study, the Cronbach's α coefficient of the scale is 0.94.

#### Work Stress

There are three common measures of work. First is using stressors that employees encountered in the workplace to reflect work stress, such as role conflict and job intensity (Rees and Smith, 1991); second is using the reaction of employees to work stress to represent work stress, such as emotional exhaustion and nervousness (Cheong et al., 2016); third is to measure the perception of employees of work stress directly (Hongli and Quanjun, 2016). The former two are proxy measures. And in this study, HPWS is the source of work stress, the well-being of the employee is the reaction of employees to work stress, it is inappropriate to measure work stress by stressors or the reaction to work stress. Therefore, we choose the third method to measure work stress, i.e., measuring work stress directly. Work stress adopted the scale used by Hongli and Quanjun (2016), with a total of three items, including “My job is extremely stressful,” “There are very few things that are not stressful at work,” and “I feel very stressful for my profession.” In this study, the Cronbach's α coefficient of the scale is 0.77.

#### The Well-Being of the Employee

We measured the well-being of the employee by using job satisfaction, emotional commitment, and emotional exhaustion (reverse scoring) to form a combined scale based on the recommendation of Koopman et al. (2016). Job satisfaction is an indicator that is commonly used to measure the well-being of the employee, reflecting the subjective evaluation of work quality by the employee or all experience at work (Ilies et al., 2007). Affective commitment is a positive emotional connection between employees and the company, reflecting the emotional experience of employees (Meyer et al., 2002). Emotional exhaustion focuses on reflecting the cognition and physiological experience of employees when facing stress in the enterprise (Fritz and Sonnentag, 2006). The sample items include “I am generally satisfied with the sense of accomplishment I get from this job,” and “Working in this company gives me a sense of belonging.” The questionnaire includes 12 items; the Cronbach's α coefficient of the scale is 0.84.

#### Union Practice

The scale of labor union practice adopted the scale developed by Mao-long et al. (2018), which includes three dimensions of coordinating labor relations, caring for the lives of employees, and carrying out cultural and recreational activities, with a total of 13 items. The sample items include “union promote the disclosure of enterprises and publicizing necessary management matters,” and “The union takes measures to care for the health of employees.” In this study, the Cronbach's α coefficient of the scale is 0.94.

### Scale Validation

This study uses the Mplus software to perform confirmatory factor analysis on the measured variables to prove that the measured variables are all different, not the same. It can be seen from Table 1 that the fit validity of the 5-factor model is significantly better than other models, so the variables measured in the study have good discriminating validity.

**Table 1:** Comparison of the goodness-of-fit indices of alternative models.

**Model**	**χ^2^**	**df**	**χ^2^/df**	**CFI**	**TLI**	**RMSEA**	**SRMR**
5 factor model	2,844.22	1,168	2.435	0.83	0.80	0.08	0.07
4 factor model	3,436.74	1,172	2.932	0.77	0.73	0.09	0.08
3 factor model	3,567.93	1,175	3.036	0.75	0.72	0.10	0.08
2 factor model	3,660.40	1,177	3.109	0.74	0.71	0.10	0.08
1 factor model	4,199.73	1,178	3.565	0.69	0.65	0.10	0.09

*Two-factor models: union practice + hpws, POS + work stress + the well-being of the employee; Three-factor models: union practice + hpws, POS + work stress, the well-being of the employee; Four-factor models: union practice + hpws, POS, work stress, the well-being of the employee. Hpws, high-performance work systems; POS, perceived organizational support*.

## Results

### Means, SD, and Correlations Among Study Variables

The means, SD, and correlation matrix of each variable in the study are shown in Table 2. Table 2 reveals that HPWS is positively correlated with organizational support (r = 0.66, *p* < 0.01), and perceived organizational support is positively correlated with the well-being of the employee (r = 0.64, *p* < 0.01), which preliminarily reflects the logic of H1. HPWS is significantly positively correlated with work stress (r = 0.17, *p* < 0.01), and work stress is significantly negatively correlated with the well-being of the employee (r = −0.23, *p* < 0.01), which initially reflects the logic of H2.

**Table 2:** Means, SD, and correlations.

	**M ± SD**	**1**	**2**	**3**	**4**
1. High-performance work systems	3.88 ± 0.72				
2. Union practice	3.94 ± 0.88	0.68**			
3. Perceived organizational support	3.76 ± 0.87	0.66**	0.48**		
4. Work stress	3.38 ± 0.85	0.17**	0.10	0.14*	
5. Employee well-being	3.59 ± 0.66	0.59**	0.47**	0.64**	−0.23**

*M, mean; SD, standard deviation; N, 243;*

** *p < 0.01*,

* *p < 0.05, below is same*.

### Hypotheses Test

**Figure 1:**
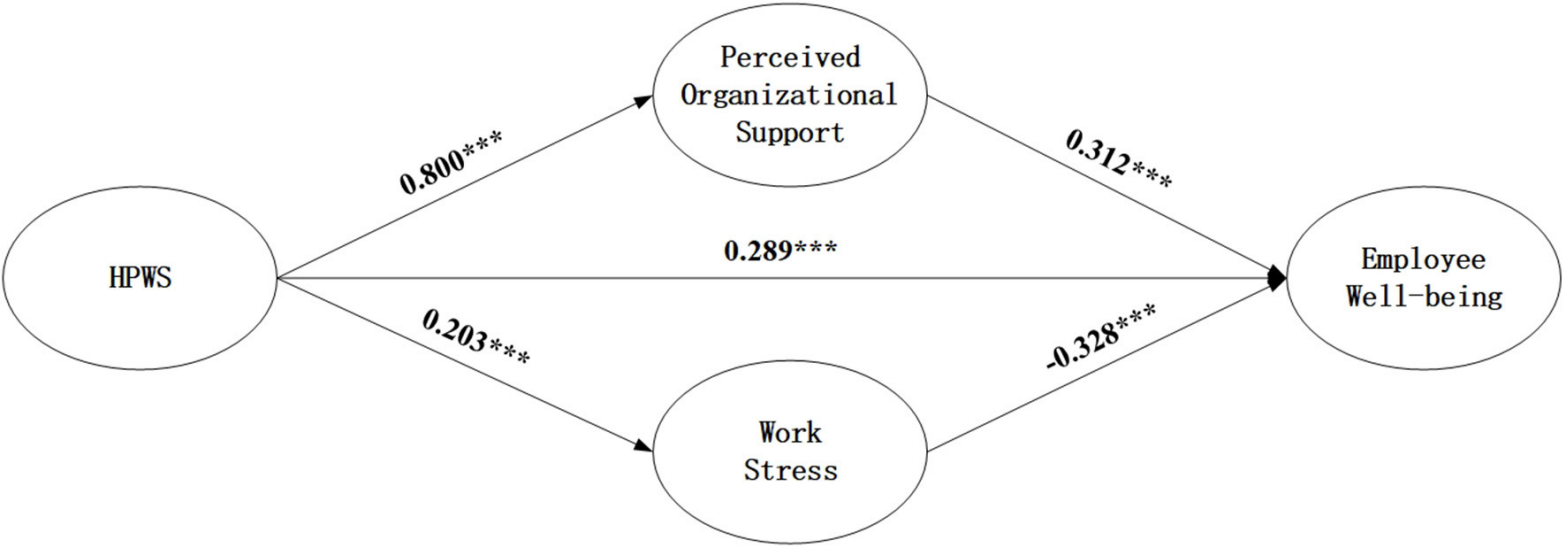
The effect of HPWS on employee well-being.

We constructed the path analysis of the proposed model using Mplus 8.3 software to confirm H1 and H2. Figure 1 shows the results of path analysis. It can be seen from Figure 1 that HPWS has a significant positive effect on perceived organizational support (β = 0.401, *p* < 0.05), and there is a significant positive relationship between perceived organizational support (β = 0.330, *p* < 0.05); HPWS has a significant positive effect on work stress (β = 0.184, *p* < 0.05), and there is a significant negative relationship between work stress and the well-being of the employee (β = −0.338, *p* < 0.05).

The mediating effect of perceived organizational support and work stress were shown in Table 3. It can be seen from Table 3 that the mediating effect of perceived organizational support between HPWS and the well-being of the employee is 0.229, and the 95% confidence interval is from 0.192 to 0.371, excluding zero in the CI, which suggest that the indirect effect of HPWS on the well-being of the employee through perceived organizational support is significant. Thus, H1c was supported. The mediating effect of work stress between HPWS and the well-being of the employee is −0.055, and the 95% confidence interval is from −0.104 to −0.010, excluding zero in the CI, which suggests that the indirect effect of HPWS on the well-being of the employee through work stress is significant. H2 was supported.

**Table 3:** Mediating effect of perceived organizational support and work stress.

**Path**	**Mediating effect**		**Confidence interval (95%)**	
	**Estimate**	**SE**	**BootLLCI**	**BootULCI**
HPWS → POS → WB	0.229	0.045	0.192	0.371
HPWS → WS → WB	−0.055	0.022	−0.104	−0.010

*HPWS, high performance systems; POS, perceived organizational support; WS, work stress*.

We first verify the moderating effect of union practice on the relationship between HPWS and perceived organizational support. We regressed perceived organizational support on HPWS, union practice, the interaction of HPWS and union practice, and demographic variables to verify the moderating effect of union practice. The same method is used when examining moderating effect of union practice on the relationship between HPWS and work stress. Model 2 in Table 4 reveals that the interaction term has no significant effect on the organizational support (β = −0.054, *p* > 0.05), which means the moderating effect of union practice on the relationship between the HPWS and perceived organizational support is not significant. Therefore, hypotheses H3 and H5 have not been supported. Model 4 shows that union practice can significantly moderate the relationship between HPWS and work stress (β = −0.281, *p* < 0.01), and H4 has been supported.

**Table 4:** Moderating effect of union practice.

	**Perceived organizational support**		**Work stress**	
	**M1**	**M2**	**M3**	**M4**
Gender	−0.14	−0.15	−0.10	−0.09
Age	−0.01	−0.01	−0.10	−0.11
Education	−0.12	−0.07	0.01	0.02
Political	−0.11	0.05	0.02	0.08
Company type	0.11	0.08	0.04	0.04
Company size	0.05	0.02	0.04	0.04
Company position	−0.10	−0.04	−0.10	−0.08
Union position	−0.09	−0.08	−0.04	−0.05
hpws		0.74***		0.19
up		0.06		−0.02
up*hpws		−0.05		−0.28**
*R^2^*	0.09	0.47	0.03	0.10
*ΔR^2^*	0.09**	0.38***	0.03	0.07**

*Up, union practices; hpws, high-performance work systems*.

** *p < 0.01*,

*** *p < 0.001*.

**Figure 2:**
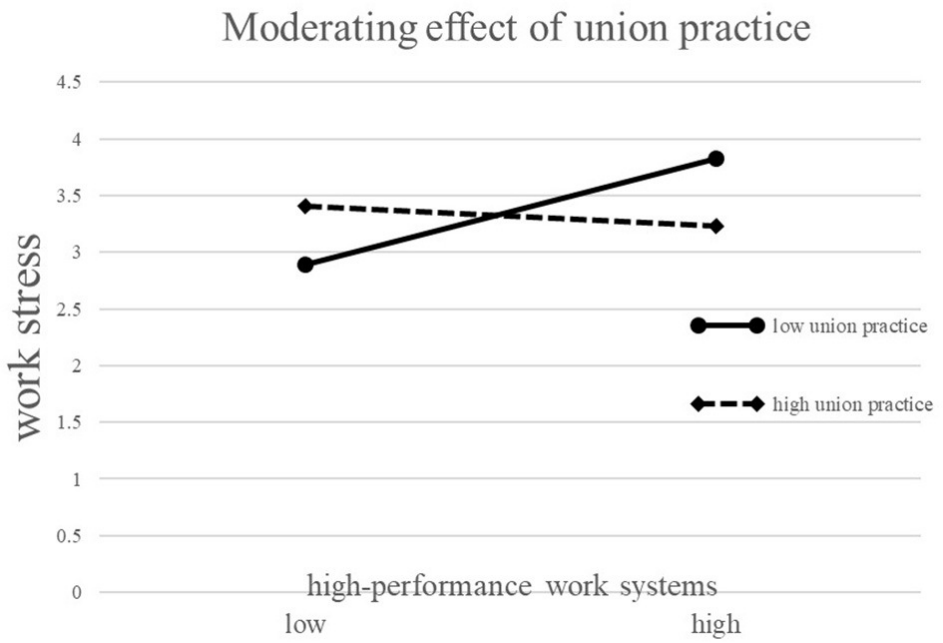
The moderating effect of union practice.

To more intuitively express the moderating effect of trade union practice between the HPWS and work stress, the study used the method of Aiken et al. (1991) to test the slope of the regression of the independent variable to the outcome variable in different groups according to union practice. Figure 2 reveals that HPWS has different effects on work stress in groups with different union practice levels. Specifically, in companies with high labor union practices, the positive relationship between HPWS and work stress is weak. In contrast, in companies with low labor union practices, the positive relationship between HPWS and work stress is stronger.

The above result shows that the trade union practice can moderate the relationship between the HPWS and work stress. In contrast, moderating effect of union practice on the relationship between the HPWS and the perceived organizational support is not significant. Therefore, we used model 7 of the process to verify the mediating effect on the relationship between HPWS and the well-being of the employee through work stress. Table 5 reveals that the 95% confidence interval of the indirect effect difference among different union practice groups does not include 0 [β = 0.133, SE = 0.055, and BootLCI = (0.032 and 0.251)], which indicates that the difference of indirect effect in different union practice group is significant. H6 has been supported, which means that union practice can significantly moderate the impact of HPWS on the well-being of the employee through work stress.

**Table 5:** Moderated mediating effect.

**Path**	**Index**	**Effect**	**BootSE**	**Boot95% CI**	
				**Low**	**High**
HPWS → WS → WB	Eff1(M-1SD)	−0.111	0.036	−0.193	−0.054
	Eff2(M)	−0.044	0.029	−0.106	0.010
	Eff3(M+1SD)	0.022	0.022	−0.062	0.109
Comparison of effect	Eff2-Eff1	0.066	0.028	0.161	0.126
	Eff3-Eff1	0.133	0.055	0.032	0.251
	Eff3-Eff2	0.066	0.028	0.161	0.126

## Discussion

This study exploredthe double-edged sword effect of HPWS on the well-being of the employee through perceived organizational support and work stress and the moderating effect of union practice through the integration of research in human resources and labor relations. This study found the following results:

First, HPWS has a double-edged sword effect on the well-being of the employee. Specifically, HPWS positively impacts the well-being of the employees by nourishing perceived organizational support; on the other hand, HPWS has a negative impact on the well-being of the employee by increasing work stress. This result echoes the proposition that HPWS has a positive and negative effect on the well-being of the employee simultaneously (Zhang et al., 2013). In addition, the results of this study also confirmed Han et al. (2020) view that the JD-R model should be used to explain the internal mechanism of how HPWS influences the well-being of the employee.

Second, union practice attenuates the positive effect of HPWS on work stress and further reduces the negative impact of HPWS on the well-being of the employees through work stress. This is consistent with the results of Chen et al. (2018) that union can moderate the relationship between HPWS and attitude and behavior of employees in China; however, union practice did not moderate the relationship between HPWS and perceived organizational support. According to Hobfoll et al. (2018), even a small loss of resources may cause failure in biological systems. From the perspective of individual psychology, the damage of resource loss is far greater than the beneficial degree of resource acquisition. Providing individuals with additional resources or other means to reduce the loss of resources will affect attitudes or behaviors of individuals stronger. In this study, the role of the union in reducing the loss of resources was perceived by employees more apparently. Therefore, union practice can significantly moderate the relationship between HPWS and work stress, whereas union practice did not moderate the relationship between HPWS and perceived organizational support.

### Theoretical Contribution

First, this study constructed a framework to understand the double-edged sword effect of HPWS on the well-being of the employee. There are two main views among previous studies: optimism believes that HPWS increases work autonomy and work participation of employees, which increase the well-being of the employees; pessimism believes that HPWS increases work requirement through strategic authorization, which reduces the well-being of the employees (Guest, 2017). Current studies have conducted preliminary discussions on the double-edged sword of HPWS (Han et al., 2020); however, the internal mechanism of the relationship between HPWS and the well-being of the employee was ambiguous, and few studies used empirical data to verify such a double-edged sword effect of HPWS. Based on the JD-R model, this study employed perceived organizational support and work stress as mediating variables to construct a framework to understand the internal mechanism of how HPWS influences the well-being of the employee and verified the double-edged sword effect of HPWS on the well-being of the employee through empirical research.

Second, this study sheds a light on and verifies the effect of unions in management in China from a more explicit aspect. With the progress of trade union reform in China, unions became an essential part of enterprise management. Chinese unions play two roles in the workplace, protecting the rights of employees and promoting production efficiency (Chen et al., 2018). Scholars held the view that unions can increase the positive effects of HRM practices due to their role in promoting production efficiency, and weaken the adverse effects of HRM practices due to their role in protecting the rights and interests of employees (Wang and Zheng, 2012); however, there is a lack of empirical research to verify the effect of the union from the perspectives of these two roles, even few research studies confirm the effect of the union in enterprise management. This study employed union practice as a moderating variable to explore its effect on the relationship between HPWS and work stress, and HPWS and perceived organizational support. These results confirmed the role of union as a facilitator of HRM and further revealed that unions act more as protectors in the workplace. This contributes to the understanding of the current management systems in enterprises with a union in China and the field of constructing a fair and effective management system in the workplace.

### Practical Implications

First, for enterprises, more attention should be paid to the process of implementing HPWS. The performance of enterprises and the well-being of the employee have always been regarded as two essential goals pursued by HRM. HPWS undoubtedly increases the performance of the enterprise (Wei and Lau, 2010); however, the results of this study showed that HPWS has a double-edged sword effect on the well-being of the employee. This indicate that if enterprise adopt HPWS out of pursuing profit totally, employees' well-being will be reduced. Therefore, enterprises should be careful in balancing enterprise performance and the well-being of the employee. Specifically, clarifying job responsibility, loosening the connection between performance and promotion or between performance and salary may be effective ways to ensure the well-being of the employees in the workplace.

Second, trade unions in China should actively exert their subjective initiative, ensuring a positive workplace experience for employees. Trade unions are embedded in enterprises in China. In recent years, with the progress of trade union reform, trade unions have gradually played a more influential role in management in enterprises in China (Chen et al., 2018); however, due to part-time personnel and economic dependence on enterprises, the work of the trade union still has problems, such as lack of work enthusiasm (Cooke, 2014). From the research results of this article, union practices can effectively help reduce the adverse effects of HPWS and play an essential role in ensuring the well-being of the employees in the workplace. Therefore, the labor union should go deep into the employee group, actively carry out various rights protection, skills competition, and other activities, achieve the coordinated development of fairness and efficiency in the workplace.

### Limitations and Suggestions for Future Research

First, this questionnaire survey was conducted at the same time point, which resulted in the measured data only reflecting the current HPWS, union practice, and the state of the well-being of the employee. Although it reflects the relationship between the variables to a certain extent, it cannot fully reflect the causal relationship between variables. In addition, the flow of resources and its impact on employees have dynamic and long-term effects (Halbesleben et al., 2014). Therefore, future research can use multiple time points to collect data to reflect the effect of HPWS on the well-being of the employee and the role of union practice in it.

Second, this research measures the HPWS and union practice at an individual level. There may be differences between the HPWS at the individual and enterprise levels in the same workplace. The HPWS at the individual level may be different due to the position of the individual, personality traits, and the environment of the department (Junwei et al., 2017). Similarly, there may be differences between trade union practices at the individual and enterprise levels. Therefore, future research should measure HPWS and union practices at both enterprise-level and individual-level to more fully reflect the influence of HPWS and union practices on the well-being of the employee.

Third, this study discusses the relationship between HPWS and the well-being of the employee based on the JD-R model, without considering the role of attribution theory and self-determination theory in explaining this issue. Theories, such as attribution theory and self-determination theory, can help researchers explain the double-edged sword effect of HPWS (Han et al., 2020). So future research can combine other theories to carry out further research on this issue.

Fourth, prior studies also show that social support within an enterprise, including support from leaders, colleagues, or family, can effectively reconcile the negative effects caused by HPWS (Guest, 2017). Therefore, future studies should integrate these factors into the analytical framework to find ways to cope with the dark side of HPWS.

## Data Availability Statement

The raw data supporting the conclusions of this article will be made available by the authors, without undue reservation.

## Ethics Statement

Ethical review and approval was not required for the study on human participants in accordance with the local legislation and institutional requirements. Written informed consent for participation was not required for this study in accordance with the national legislation and the institutional requirements.

## Author Contributions

WQ contribute to data collection, data analysis, and writing. HE, SJ, and SH reviewed the manuscript and give the suggestions.

## Conflict of Interest

The authors declare that the research was conducted in the absence of any commercial or financial relationships that could be construed as a potential conflict of interest.

## Publisher's Note

All claims expressed in this article are solely those of the authors and do not necessarily represent those of their affiliated organizations, or those of the publisher, the editors and the reviewers. Any product that may be evaluated in this article, or claim that may be made by its manufacturer, is not guaranteed or endorsed by the publisher.
